# Synthesis, crystal structure and Hirshfeld surface of ethyl 2-[2-(methyl­sulfan­yl)-5-oxo-4,4-diphenyl-4,5-di­hydro-1*H*-imidazol-1-yl]acetate (thio­phenytoin derivative)

**DOI:** 10.1107/S2056989024007345

**Published:** 2024-08-09

**Authors:** Abderrazzak El Moutaouakil Ala Allah, Benson M. Kariuki, Abdulsalam Alsubari, Ahlam I. Al-Sulami, Basmah H. Allehyani, Wafa O. Alsulami, Joel T. Mague, Youssef Ramli

**Affiliations:** a Laboratory of Medicinal Chemistry Drug Sciences Research Center Faculty of Medicine and Pharmacy Mohammed V University in Rabat Morocco; b School of Chemistry, Cardiff University, Main Building, Park Place, Cardiff, CF10 3AT, United Kingdom; c Laboratory of Medicinal Chemistry, Faculty of Clinical Pharmacy, 21 September University, Yemen; d University of Jeddah, College of Science, Department of Chemistry, Jeddah 21589, Saudi Arabia; e Department of Chemistry, Tulane University, New Orleans, LA, 70118, USA; Venezuelan Institute of Scientific Research, Venezuela

**Keywords:** crystal structure, thio­phenytoin, di­hydro­imidazole, hydrogen bond, ester, thio­ether

## Abstract

The di­hydro­imidazole ring in the title mol­ecule, C_20_H_20_N_2_O_3_S, is slightly distorted and the lone pair on the tri-coordinate nitro­gen atom is involved in intra-ring π bonding. The methyl­sulfanyl substituent lies nearly in the plane of the five-membered ring while the ester substituent is rotated well out of that plane. In the crystal, C—H⋯O hydrogen bonds form inversion dimers, which are connected along the *a*- and *c-*axis directions by additional C—H⋯O hydrogen bonds, forming layers parallel to the *ac* plane. The major contributors to the Hirshfeld surface are C⋯H/H⋯C, O⋯H/H⋯O and S⋯H/H⋯S contacts at 20.5%, 14.7% and 4.9%, respectively.

## Abstract 2

The di­hydro­imidazole ring in the title mol­ecule is slightly distorted and the lone pair on the tri-coordinate nitro­gen atom is involved in intra-ring π bonding. In the crystal, C—H⋯O hydrogen bonds form inversion dimers, which are connected along the *a*- and *c-*axis directions by additional C—H⋯O hydrogen bonds, forming layers parallel to the *ac* plane.

## 1. Chemical context

**Figure 1:**
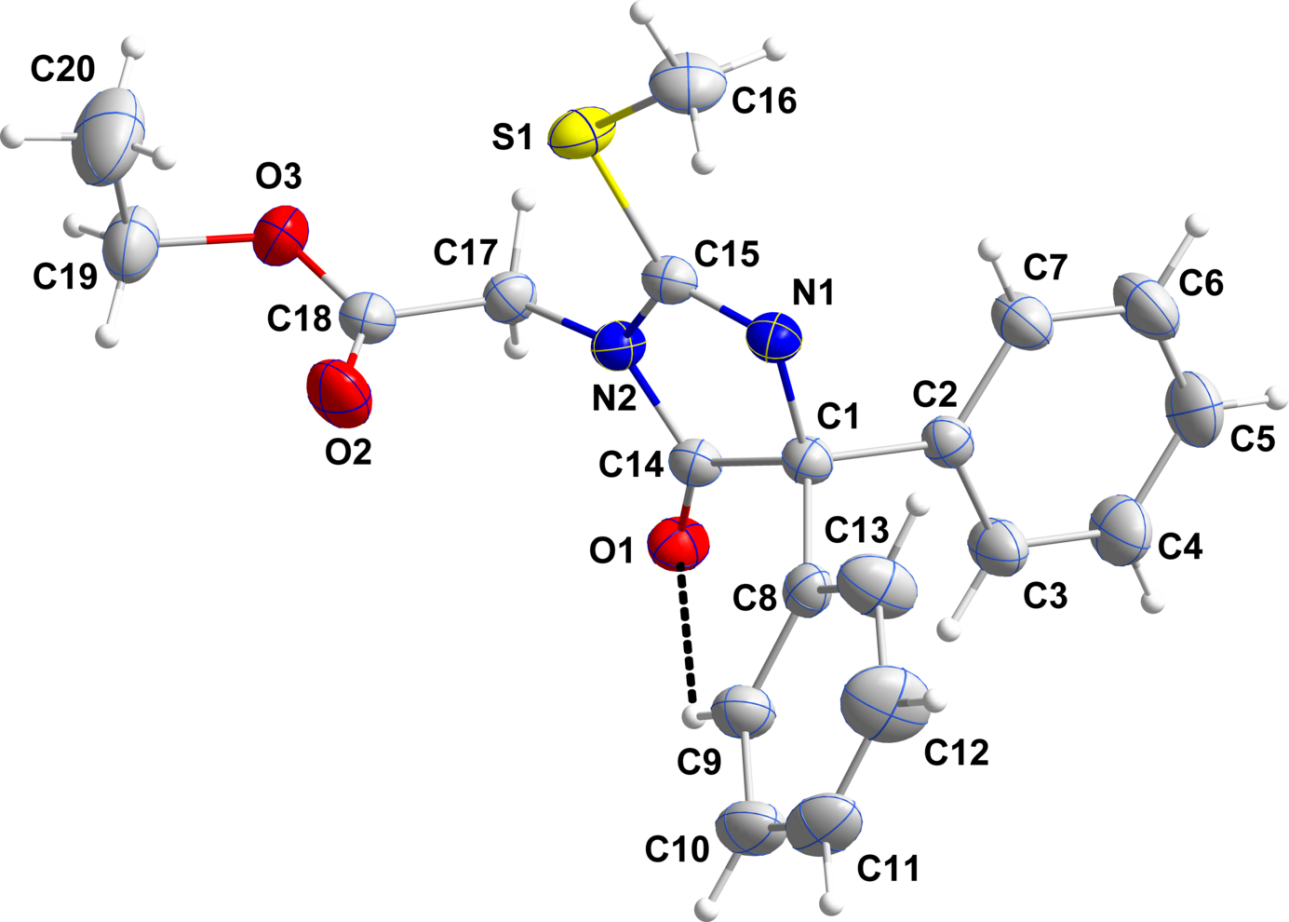
The title mol­ecule with the labeling scheme and 30% probability ellipsoids. The intra­molecular C—H⋯O hydrogen bond is depicted by a dashed line and only the major component of the disorder is shown.

The family of hydantoin drugs is important in medicinal chemistry because of the wide range of pharmacological activities exhibited, including anti­bacterial, anti­diabetic, anti-inflammatory, anti­convulsant, anti-HIV and anti­cancer properties. Thio­hydantoins, sulfur analogues of hydantoins, undergo replacement of one or both carbonyl groups with thio­carbonyl groups. This substitution enables versatile structural modifications, facilitating the customization of thio­hydantoins to preferentially adopt specific structural types. Such modifications, achieved by introducing steric bulk, altering hydro­philic or hydro­phobic inter­actions, or promoting stacking, afford control over the mol­ecule’s ability to form hydrogen-bonded arrays in the solid state. In particular, phenytoin and thio­phenytoin derivatives and diphenyl-substituted hydantoin exhibit significant activity against tonic–clonic (grand mal) seizures (Camerman & Camerman, 1971 ▸). These chemicals are recognized for their anti­convulsant properties and have diverse pharmacological applications, including anti­fungal, herbicidal, anti-inflammatory, anti-HIV, anti­microbial, anti­cancer, and anti­bacterial activities, which vary based on the specific substitutions on the hydantoin ring (Cho *et al.*, 2019 ▸; Allah *et al.*, 2024 ▸; El Moutaouakil Ala Allah *et al.*, 2024*a* ▸). The significance of this scaffold in drug discovery is underscored by several clinically used medications, including phenytoin, nitro­furan­toin, and enzalutamide (Patocka *et al.*, 2020 ▸). Given the wide range of therapeutic applications for such compounds, we have previously reported a route for the preparation of thio­phenytoine derivatives using N-alkyl­ation reactions carried out with ethyl bromo­acetate (Guerrab *et al.*, 2020 ▸, 2022 ▸; Missioui *et al.* 2022 ▸). A similar approach yielded the title compound, C_20_H_20_N_2_O_3_S (Fig. 1 ▸). In addition to the synthesis, we also report the mol­ecular and crystal structure along with a Hirshfeld surface analysis.
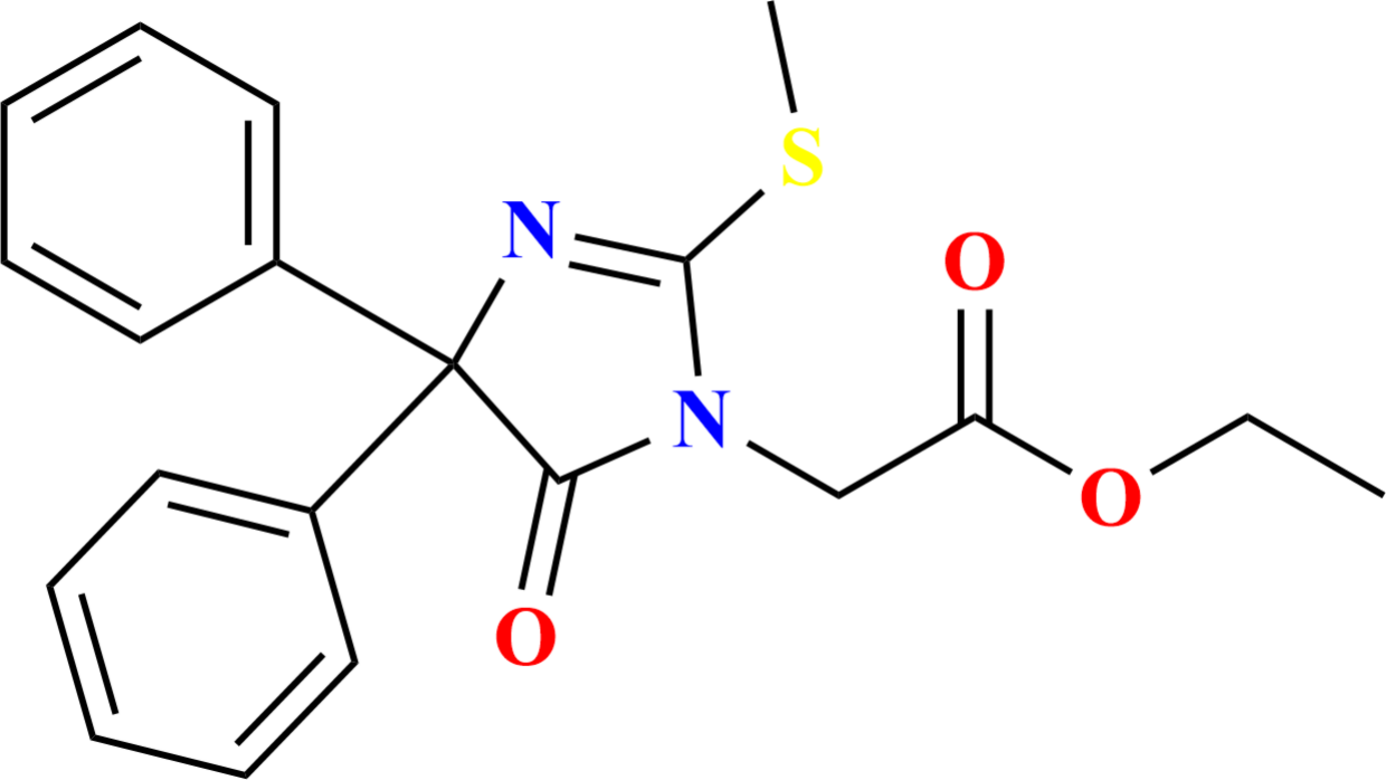


## 2. Structural commentary

**Figure 2:**
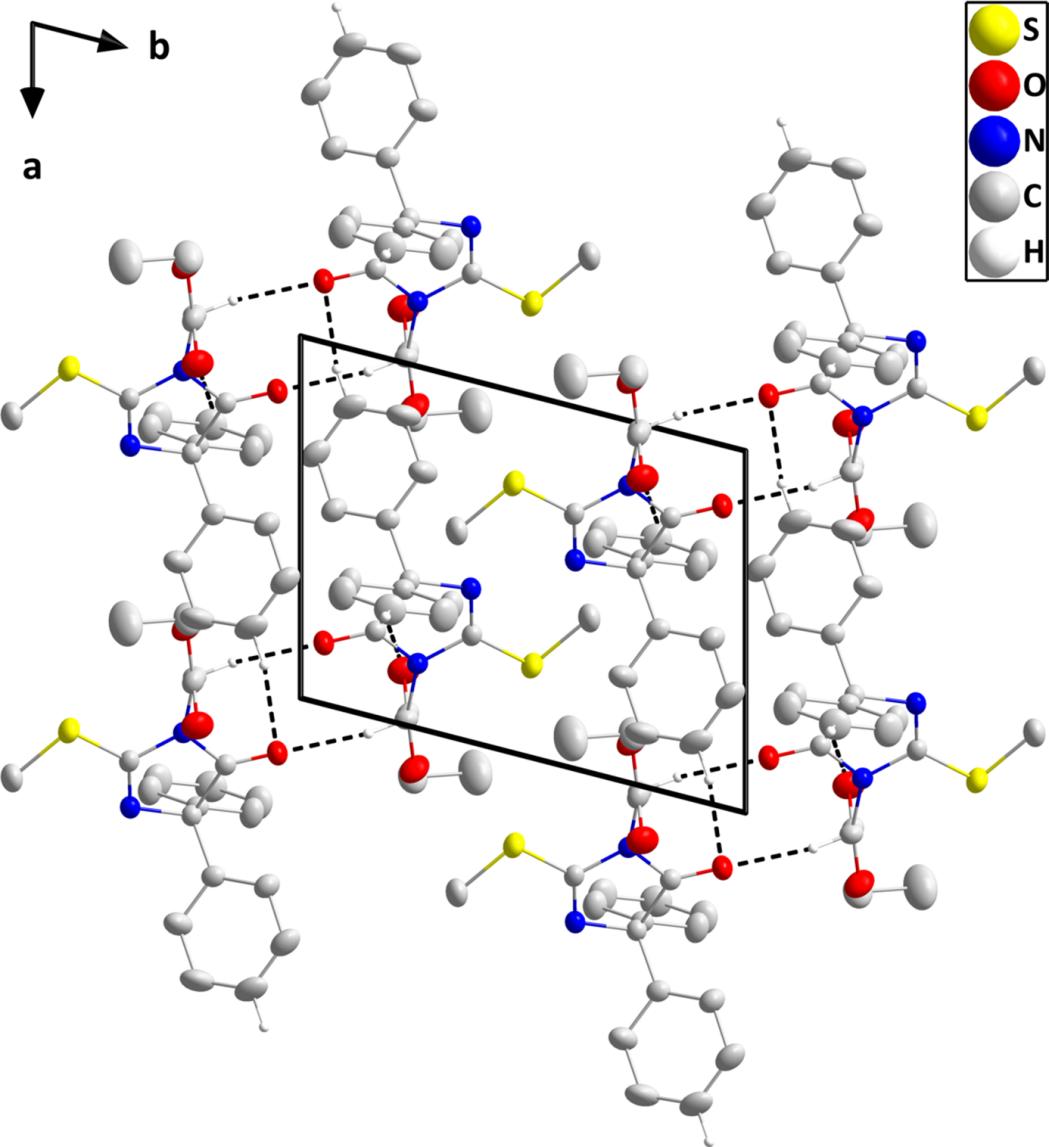
Packing viewed along the *c*-axis direction giving end views of two adjacent layers. The C—H⋯O hydrogen bonds are depicted by dashed lines and non-inter­acting hydrogen atoms are omitted for clarity.

The di­hydro­imidazole ring is slightly distorted with C1 located 0.0166 (10) Å to one side of the mean plane and C14 positioned 0.0199 (10) Å on the other side (r.m.s. deviation of the fitted atoms = 0.0143 Å). The ethoxy group is disordered over two resolved sites in a 0.741 (7)/0.259 (6) ratio. The sum of the angles about N2 is 359.89 (15)°, indicating participation of its lone pair in N→C π bonding. This occurs to a slightly greater extent with C14 as the N2—C14 bond length is 1.371 (2) Å while the N2—C15 bond length is 1.406 (2) Å. By contrast, the N2—C17 bond length is 1.445 (2) Å. The C16 methyl group lies nearly in the plane of the di­hydro­imidazole ring as the C16—S1—C15—N2 torsion angle is −176.94 (14)° but the ester substituent is directed well out of this plane since the C15—N2—C17—C18 torsion angle is −76.6 (2)° (Fig. 2 ▸). The rotational orientation of the C8⋯C13 phenyl ring is partially determined by the intra­molecular C9—H9⋯O1 hydrogen bond (Fig. 2 ▸).

## 3. Supra­molecular features

In the crystal, inversion dimers are formed by paired C17—H17*A*⋯O1 hydrogen bonds. The dimers are formed into chains extending along the *c*-axis direction by weak C5—H5⋯O2 hydrogen bonds and the chains are linked by weak C11—H11⋯O1 hydrogen bonds (Table 1 ▸) along the *a*-axis direction into layers parallel to the the *ac* plane. The layers pack with normal van der Waals contacts (Fig. 2 ▸).

## 4. Database survey

**Figure 3:**
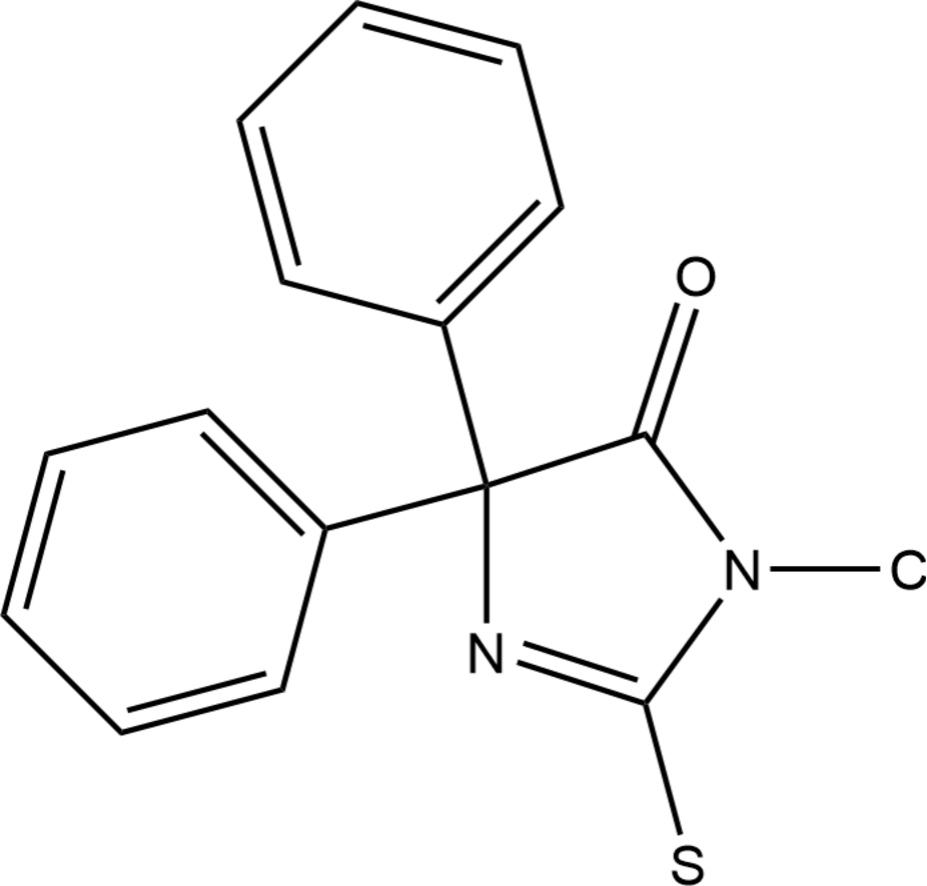
The search fragment used for the database survey.

A search of the Cambridge Structural Database (CSD; updated to May 31, 2024; Groom *et al.*, 2016 ▸) with the fragment shown in Fig. 3 ▸ yielded ten hits including those with methyl (YEYYA; El Moutaouakil Ala Allah *et al.*, 2023 ▸), ethyl (HOPQAI; Allah *et al.*, 2024*a* ▸), *n*-propyl (RIJZIW: Akrad *et al.*, 2018 ▸), benzyl (RAHGUF; Akrad *et al.*, 2017 ▸) and allyl (ROLJAH; El Moutaouakil Ala Allah *et al.*, 2024*b* ▸) subs­tituents on both nitro­gen and sulfur. The remainder have the nitro­gen and sulfur connected by a —CH_2_CH_2_— chain (DIYRAE; El Moutaouakil Ala Allah *et al.*, 2023 ▸), a —CH_2_CH(COOEt)— chain (FURFED; Karolak-Wojciechowska & Kiec-Kononowicz, 1987 ▸), a —CH_2_CH_2_CH_2_— chain (IMTHZN; Kieć-Kononowicz *et al.*, 1981 ▸ and IMTHZN01; Guerrab *et al.*, 2019 ▸) and a —CH_2_CH_2_(OCH_2_CH_2_)_2_OCH_2_CH_2_— chain (LIGWOR; Guerrab *et al.*, 2023 ▸). In all cases, the di­hydro­imidazole ring is planar with the maximum deviation of a fitted atom from the mean plane ranging from 0.006 (1) Å (HOPQAI, r.m.s. deviation of the fitted atoms = 0.001 Å) to 0.023 (2) Å (RAHGUF, r.m.s. deviation of the fitted atoms = 0.002 Å) for those not have a ring fused to it and up to 0.029 (2) Å (IMTHZN01, r.m.s. deviation of the fitted atoms = 0.002 Å) where a fused ring is present. Particularly where the second ring size is relatively small (DIYRAE, FURFED, IMTHZN and IMTHZN01), it is likely that strain from the ring fusion contributes to the greater deviation from planarity. With the exception of the four just mentioned where geometrical constraints require it, all structures have the carbon attached to sulfur in the side chain very close to the mean plane of the di­hydro­imidazole ring as in the title mol­ecule. The same group of structures has the β-carbon of the substituent on nitro­gen oriented well out of that plane.

## 5. Hirshfeld surface analysis

**Figure 4:**
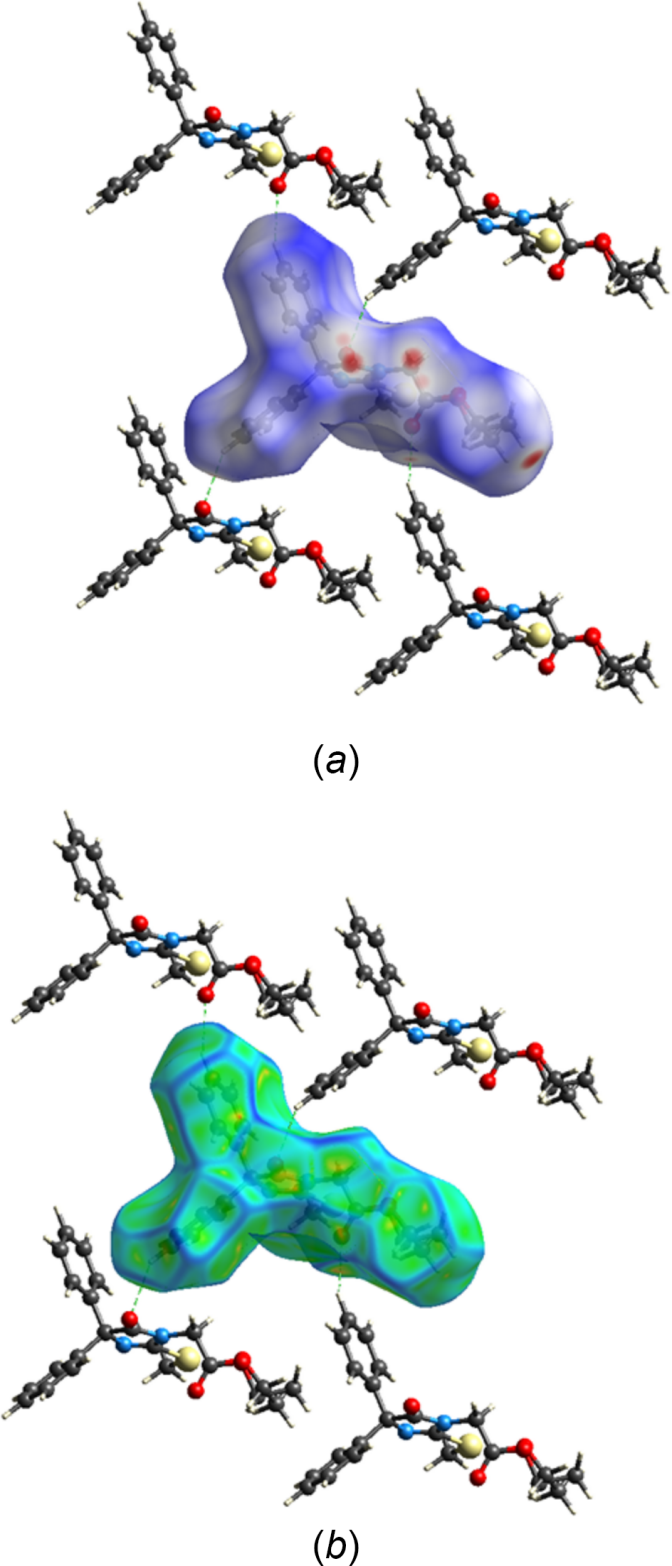
The Hirshfeld surface plotted over (*a*) *d*_norm_ and (*b*) curvature with four neighboring mol­ecules. C—H⋯O hydrogen bonds are depicted by green dashed lines.

**Figure 5:**
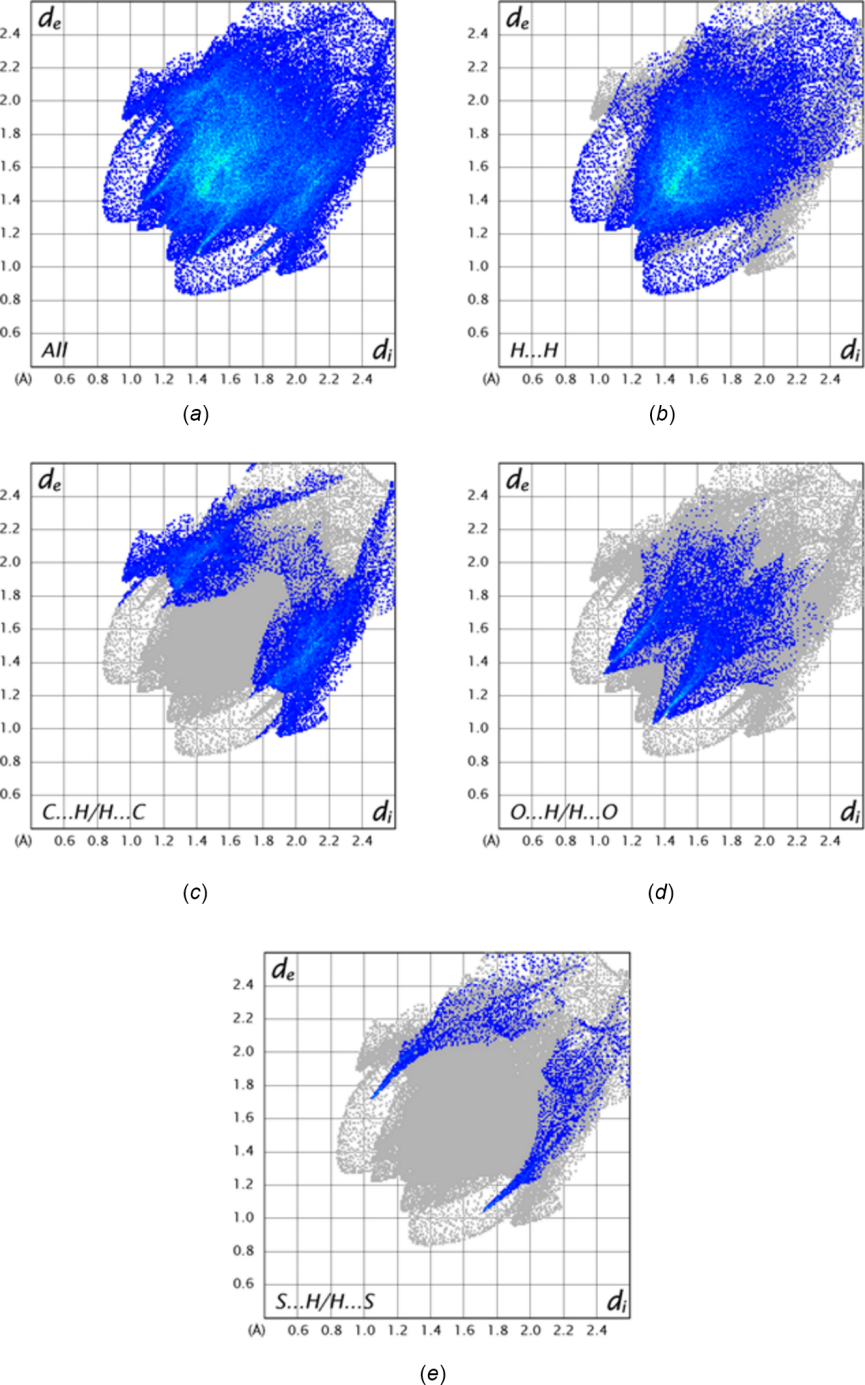
Fingerprint plots showing (*a*) all inter­molecular contacts and delineated into (*b*) H⋯H, (*c*) C⋯H/H⋯C, (*d*) O⋯H/H⋯O contacts and (*e*) S⋯H/H⋯O contacts.

A Hirshfeld surface analysis of the inter­molecular inter­actions of the title mol­ecule was carried out with *Crystal Explorer 21.5* (Spackman *et al.*, 2021 ▸) and descriptions of the graphical output and its inter­pretation have been published (Tan *et al.*, 2019 ▸). The *d*_norm_ surface calculated over the range −0.2373 to 1.3807 in arbitrary units is shown in Fig. 4 ▸*a* together with four neighboring mol­ecules illustrating the weak C5—H5⋯O2 and C11—H11⋯O1 hydrogen bonds while Fig. 4 ▸*b* shows the surface calculated over the curvature function. The latter shows that there are no extensive flat regions about the mol­ecule, consistent with the absence of π-stacking inter­actions. Fig. 5 ▸ shows the 2-D fingerprint plot of all inter­molecular inter­actions and those delineated into contributions from H⋯H, C⋯H/H⋯C, O⋯H/H⋯O and S⋯H/H⋯S inter­actions. Here, the H⋯H inter­actions comprise 56.4% of the total, consistent with the high hydrogen content of the mol­ecule and the shape, which has many of the hydrogen atoms pointing outwards from the center of gravity. The other significant contributions are from C⋯H/H⋯C, O⋯H/H⋯O and S⋯H/H⋯S contacts at 20.5%, 14.7% and 4.9%, respectively.

## 6. Synthesis and crystallization

**Figure 6:**
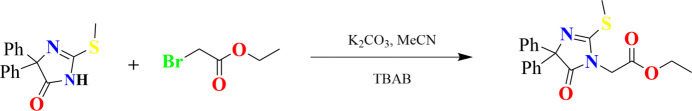
Synthesis of the title compound.

To a solution of 2-(methyl­sulfan­yl)-5,5-diphenyl-3,5-di­hydro-4*H*-imidazol-4-one (0.5 g, 1.78 mmol) in aceto­nitrile (15 mL) were added K_2_CO_3_ (0.3 g, 2 mmol) and ethyl bromo­acetate (0.19 ml, 1.80 mmol) and a catalytic qu­antity of tetra-*n*-butyl­ammonium bromide. The reaction scheme is shown in Fig. 6 ▸. The mixture was stirred for 8 h at room temperature. The solution was filtered and the solvent removed under reduced pressure. The solid obtained upon solvent removal was recrystallized from ethanol to afford thick, colorless, plate-like crystals of the title compound. Yield = 92%, m.p. = 515–517 K. **FT-IR** (ATR, υ, cm^−1^): 3082 (N—H), 3060 (H—C=C), 1731 (C=O), 1587, 1570, 1491, 1412 (Ar—C=C); **^1^H NMR** (500 MHz, CDCl_3_): δ ppm 1.24 (*t*, 3H, —O—CH_2_—C**H**_3_), 2.69 (*s*, 3H, S—C**H**_3_), 4.22 (*q*, 2H, —O—C**H_2_**—CH_3_), 4.27 (*s*, 2H, N—C**H**_2_), 7.25–7.54 (*m*, 10H, Ar—**H**); **^13^C NMR**: 12,93 (—O—CH_2_—**C**H_3_), 14.16 (—S—**C**H_3_), 41.75 (—N—**C**H_3_), 62.17 (—O—**C**H_2_—CH_3_), 78.98 (**C**—2Ph), 127.22, 128.31, 128.76, 140.14 (**C**—Ar); 160.71 (**C**=N); 167.00 (**C**=O), 180.73(**C**=O_imidazole_). **HRMS** (ESI): calculated for C_20_H_20_N_2_O_2_S [*M* − H]^+^ 369,1195; found 369,12579.

## 7. Refinement

Crystal data, data collection and structure refinement details are summarized in Table 2 ▸. Hydrogen atoms were included as riding contributions in idealized positions with isotropic displacement parameters tied to those of the attached atoms. The eth­oxy group is disordered over two sites in a 0.741 (7)/0.259 (6) ratio. The two components were refined with restraints to make their geometries be comparable.

## Supplementary Material

Crystal structure: contains datablock(s) I, global. DOI: 10.1107/S2056989024007345/zn2037sup1.cif

Structure factors: contains datablock(s) I. DOI: 10.1107/S2056989024007345/zn2037Isup3.hkl

Supporting information file. DOI: 10.1107/S2056989024007345/zn2037Isup3.cml

CCDC reference: 2372876

Additional supporting information:  crystallographic information; 3D view; checkCIF report

## Figures and Tables

**Table 1:** Hydrogen-bond geometry (Å, °)

*D*—H⋯*A*	*D*—H	H⋯*A*	*D*⋯*A*	*D*—H⋯*A*
C5—H5⋯O2^i^	0.93	2.63	3.502 (3)	156
C9—H9⋯O1	0.93	2.40	3.063 (3)	128
C11—H11⋯O1^ii^	0.93	2.63	3.464 (3)	150
C17—H17*A*⋯O1^iii^	0.97	2.45	3.185 (2)	132

Symmetry codes: (i) 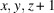; (ii) 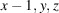; (iii) 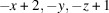.

**Table 2:** Experimental details

Crystal data	
Chemical formula	C_20_H_20_N_2_O_3_S
*M*_r_	368.44
Crystal system, space group	Triclinic, *P* 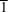
Temperature (K)	293
*a*, *b*, *c* (Å)	8.5473 (7), 10.4653 (10), 11.5360 (9)
α, β, γ (°)	88.258 (7), 74.622 (7), 75.593 (7)
*V* (Å^3^)	962.96 (15)
*Z*	2
Radiation type	Mo *K*α
μ (mm^−1^)	0.19
Crystal size (mm)	0.57 × 0.32 × 0.17

Data collection	
Diffractometer	SuperNova, Dual, Cu at home/near, Atlas
Absorption correction	Gaussian (*CrysAlis PRO*; Rigaku OD, 2023 ▸)
*T*_min_, *T*_max_	0.592, 1.000
No. of measured, independent and observed [*I* > 2σ(*I*)] reflections	7781, 4523, 3377
*R*_int_	0.019
(sin θ/λ)_max_ (Å^−1^)	0.699

Refinement	
*R*[*F*^2^ > 2σ(*F*^2^)], *wR*(*F*^2^), *S*	0.050, 0.137, 1.09
No. of reflections	4523
No. of parameters	257
No. of restraints	106
H-atom treatment	H-atom parameters constrained
Δρ_max_, Δρ_min_ (e Å^−3^)	0.24, −0.33

Computer programs: *CrysAlis PRO* (Rigaku OD, 2023 ▸), *SHELXT* (Sheldrick, 2015 ▸), *SHELXL2019/1* (Lübben *et al.*, 2019 ▸) and *DIAMOND* (Brandenburg & Putz, 2012 ▸).

## supplementary crystallographic information

### Ethyl 2-[2-(methylsulfanyl)-5-oxo-4,4-diphenyl-4,5-dihydro-1H-imidazol-1-yl]acetate Crystal data

**Table:**

C_20_H_20_N_2_O_3_S	*Z* = 2
*M**_r_* = 368.44	*F*(000) = 388
Triclinic, *P*1	*D*_x_ = 1.271 Mg m^−^^3^
*a* = 8.5473 (7) Å	Mo *K*α radiation, λ = 0.71073 Å
*b* = 10.4653 (10) Å	Cell parameters from 3430 reflections
*c* = 11.5360 (9) Å	θ = 3.9–27.4°
α = 88.258 (7)°	µ = 0.19 mm^−^^1^
β = 74.622 (7)°	*T* = 293 K
γ = 75.593 (7)°	Block, colourless
*V* = 962.96 (15) Å^3^	0.57 × 0.32 × 0.17 mm

### Ethyl 2-[2-(methylsulfanyl)-5-oxo-4,4-diphenyl-4,5-dihydro-1H-imidazol-1-yl]acetate Data collection

**Table:**

SuperNova, Dual, Cu at home/near, Atlas diffractometer	3377 reflections with *I* > 2σ(*I*)
ω scans	*R*_int_ = 0.019
Absorption correction: gaussian (CrysAlisPro; Rigaku OD, 2023)	θ_max_ = 29.8°, θ_min_ = 3.5°
*T*_min_ = 0.592, *T*_max_ = 1.000	*h* = −11→11
7781 measured reflections	*k* = −12→13
4523 independent reflections	*l* = −15→14

### Ethyl 2-[2-(methylsulfanyl)-5-oxo-4,4-diphenyl-4,5-dihydro-1H-imidazol-1-yl]acetate Refinement

**Table:**

Refinement on *F*^2^	106 restraints
Least-squares matrix: full	Hydrogen site location: inferred from neighbouring sites
*R*[*F*^2^ > 2σ(*F*^2^)] = 0.050	H-atom parameters constrained
*wR*(*F*^2^) = 0.137	*w* = 1/[σ^2^(*F*_o_^2^) + (0.052*P*)^2^ + 0.2326*P*] where *P* = (*F*_o_^2^ + 2*F*_c_^2^)/3
*S* = 1.09	(Δ/σ)_max_ < 0.001
4523 reflections	Δρ_max_ = 0.24 e Å^−^^3^
257 parameters	Δρ_min_ = −0.32 e Å^−^^3^

### Ethyl 2-[2-(methylsulfanyl)-5-oxo-4,4-diphenyl-4,5-dihydro-1H-imidazol-1-yl]acetate Special details

**Table:**

Geometry. All esds (except the esd in the dihedral angle between two l.s. planes) are estimated using the full covariance matrix. The cell esds are taken into account individually in the estimation of esds in distances, angles and torsion angles; correlations between esds in cell parameters are only used when they are defined by crystal symmetry. An approximate (isotropic) treatment of cell esds is used for estimating esds involving l.s. planes.

### Ethyl 2-[2-(methylsulfanyl)-5-oxo-4,4-diphenyl-4,5-dihydro-1H-imidazol-1-yl]acetate Fractional atomic coordinates and isotropic or equivalent isotropic displacement parameters (Å^2^)

**Table:**

	*x*	*y*	*z*	*U*_iso_*/*U*_eq_	Occ. (<1)
C1	0.5979 (2)	0.24490 (16)	0.72577 (15)	0.0416 (4)	
C2	0.6264 (2)	0.23182 (18)	0.85255 (15)	0.0442 (4)	
C3	0.6478 (3)	0.1096 (2)	0.90440 (19)	0.0633 (6)	
H3	0.639895	0.036969	0.863850	0.076*	
C4	0.6808 (4)	0.0945 (3)	1.0157 (2)	0.0769 (7)	
H4	0.695641	0.011916	1.049348	0.092*	
C5	0.6917 (3)	0.2012 (3)	1.0767 (2)	0.0768 (7)	
H5	0.714370	0.191190	1.151414	0.092*	
C6	0.6691 (4)	0.3215 (3)	1.0269 (2)	0.0821 (8)	
H6	0.674981	0.394015	1.068756	0.099*	
C7	0.6372 (3)	0.3383 (2)	0.91425 (19)	0.0639 (6)	
H7	0.623416	0.421097	0.880978	0.077*	
C8	0.4451 (2)	0.19605 (18)	0.72080 (15)	0.0449 (4)	
C9	0.4566 (3)	0.0756 (2)	0.67024 (19)	0.0609 (5)	
H9	0.561486	0.021073	0.634043	0.073*	
C10	0.3136 (3)	0.0344 (3)	0.6726 (2)	0.0747 (7)	
H10	0.323486	−0.046878	0.637062	0.090*	
C11	0.1604 (3)	0.1110 (3)	0.7260 (2)	0.0785 (7)	
H11	0.065117	0.081614	0.729605	0.094*	
C12	0.1461 (3)	0.2321 (3)	0.7747 (3)	0.0995 (10)	
H12	0.040598	0.285935	0.810314	0.119*	
C13	0.2884 (3)	0.2752 (3)	0.7712 (2)	0.0787 (7)	
H13	0.277336	0.358453	0.803302	0.094*	
C14	0.7617 (2)	0.16849 (17)	0.63784 (15)	0.0426 (4)	
C15	0.7038 (2)	0.38504 (17)	0.60131 (16)	0.0437 (4)	
C16	0.5733 (3)	0.6489 (2)	0.5983 (2)	0.0719 (6)	
H16A	0.470718	0.622454	0.608740	0.108*	
H16B	0.568596	0.727851	0.553355	0.108*	
H16C	0.588071	0.665361	0.675577	0.108*	
C17	0.9826 (2)	0.23788 (19)	0.47982 (16)	0.0484 (4)	
H17A	1.049766	0.152095	0.493094	0.058*	
H17B	1.039754	0.303695	0.491716	0.058*	
C18	0.9700 (3)	0.24075 (19)	0.35239 (17)	0.0532 (5)	
C19	1.1395 (8)	0.2528 (5)	0.1481 (3)	0.0862 (14)	0.741 (7)
H19A	1.075894	0.198513	0.123746	0.103*	0.741 (7)
H19B	1.257026	0.219412	0.106622	0.103*	0.741 (7)
C20	1.0784 (9)	0.3934 (5)	0.1203 (4)	0.127 (2)	0.741 (7)
H20A	0.962396	0.425004	0.162843	0.190*	0.741 (7)
H20B	1.090623	0.400299	0.035378	0.190*	0.741 (7)
H20C	1.142746	0.445500	0.144878	0.190*	0.741 (7)
O3	1.1147 (2)	0.25222 (17)	0.27909 (13)	0.0738 (5)	0.741 (7)
C19A	1.0825 (18)	0.2742 (19)	0.1595 (7)	0.084 (3)	0.259 (7)
H19C	0.975229	0.336314	0.165521	0.101*	0.259 (7)
H19D	1.083765	0.192018	0.122213	0.101*	0.259 (7)
C20A	1.223 (2)	0.3291 (17)	0.0899 (9)	0.117 (4)	0.259 (7)
H20D	1.202603	0.419795	0.114703	0.176*	0.259 (7)
H20E	1.230224	0.323440	0.005607	0.176*	0.259 (7)
H20F	1.326004	0.279434	0.104436	0.176*	0.259 (7)
O3A	1.1147 (2)	0.25222 (17)	0.27909 (13)	0.0738 (5)	0.259 (7)
N1	0.57653 (19)	0.38291 (14)	0.68826 (13)	0.0450 (3)	
N2	0.82123 (18)	0.26296 (14)	0.56738 (13)	0.0453 (3)	
O1	0.83139 (17)	0.05214 (12)	0.63157 (12)	0.0551 (3)	
O2	0.8505 (2)	0.23180 (18)	0.32206 (14)	0.0776 (5)	
S1	0.74520 (7)	0.52037 (5)	0.51841 (5)	0.06199 (18)	

### Ethyl 2-[2-(methylsulfanyl)-5-oxo-4,4-diphenyl-4,5-dihydro-1H-imidazol-1-yl]acetate Atomic displacement parameters (Å^2^)

**Table:**

	*U*^11^	*U*^22^	*U*^33^	*U*^12^	*U*^13^	*U*^23^
C1	0.0425 (9)	0.0385 (9)	0.0441 (9)	−0.0092 (7)	−0.0129 (7)	0.0006 (7)
C2	0.0400 (9)	0.0484 (10)	0.0452 (9)	−0.0112 (7)	−0.0127 (7)	−0.0016 (7)
C3	0.0890 (16)	0.0551 (12)	0.0570 (12)	−0.0222 (11)	−0.0349 (11)	0.0072 (9)
C4	0.105 (2)	0.0726 (15)	0.0621 (14)	−0.0208 (14)	−0.0408 (14)	0.0151 (11)
C5	0.0902 (18)	0.0946 (19)	0.0543 (13)	−0.0213 (15)	−0.0359 (12)	0.0037 (12)
C6	0.110 (2)	0.0796 (17)	0.0719 (15)	−0.0264 (15)	−0.0445 (15)	−0.0138 (13)
C7	0.0821 (15)	0.0544 (12)	0.0632 (13)	−0.0175 (11)	−0.0319 (11)	−0.0033 (9)
C8	0.0445 (9)	0.0531 (10)	0.0407 (9)	−0.0151 (8)	−0.0154 (7)	0.0068 (7)
C9	0.0598 (12)	0.0613 (13)	0.0682 (13)	−0.0255 (10)	−0.0180 (10)	−0.0009 (10)
C10	0.0823 (18)	0.0812 (16)	0.0827 (16)	−0.0451 (14)	−0.0372 (14)	0.0096 (13)
C11	0.0690 (16)	0.103 (2)	0.0905 (17)	−0.0454 (15)	−0.0465 (14)	0.0271 (15)
C12	0.0446 (13)	0.113 (2)	0.139 (3)	−0.0153 (14)	−0.0239 (15)	−0.011 (2)
C13	0.0483 (12)	0.0789 (16)	0.109 (2)	−0.0124 (11)	−0.0215 (12)	−0.0191 (14)
C14	0.0427 (9)	0.0418 (9)	0.0459 (9)	−0.0120 (7)	−0.0145 (7)	0.0002 (7)
C15	0.0458 (10)	0.0384 (9)	0.0513 (10)	−0.0121 (7)	−0.0189 (8)	0.0023 (7)
C16	0.0719 (15)	0.0458 (11)	0.0958 (17)	−0.0059 (10)	−0.0274 (13)	0.0069 (11)
C17	0.0427 (9)	0.0521 (10)	0.0502 (10)	−0.0147 (8)	−0.0089 (8)	−0.0004 (8)
C18	0.0612 (12)	0.0475 (10)	0.0531 (11)	−0.0201 (9)	−0.0123 (9)	−0.0012 (8)
C19	0.100 (3)	0.087 (3)	0.055 (2)	−0.020 (2)	0.0046 (19)	0.0016 (18)
C20	0.160 (5)	0.106 (4)	0.087 (3)	−0.009 (3)	−0.013 (3)	0.030 (3)
O3	0.0843 (11)	0.0866 (11)	0.0528 (8)	−0.0398 (9)	−0.0054 (8)	0.0096 (7)
C19A	0.096 (5)	0.094 (5)	0.053 (4)	−0.028 (4)	0.001 (4)	0.002 (4)
C20A	0.119 (8)	0.145 (8)	0.075 (6)	−0.042 (7)	0.003 (6)	0.023 (6)
O3A	0.0843 (11)	0.0866 (11)	0.0528 (8)	−0.0398 (9)	−0.0054 (8)	0.0096 (7)
N1	0.0456 (8)	0.0386 (8)	0.0510 (8)	−0.0081 (6)	−0.0158 (7)	0.0022 (6)
N2	0.0427 (8)	0.0409 (8)	0.0508 (8)	−0.0112 (6)	−0.0090 (7)	0.0014 (6)
O1	0.0547 (8)	0.0396 (7)	0.0646 (8)	−0.0063 (6)	−0.0097 (6)	0.0000 (6)
O2	0.0765 (11)	0.0972 (13)	0.0711 (10)	−0.0292 (9)	−0.0318 (9)	−0.0105 (9)
S1	0.0646 (3)	0.0443 (3)	0.0763 (4)	−0.0191 (2)	−0.0135 (3)	0.0125 (2)

### Ethyl 2-[2-(methylsulfanyl)-5-oxo-4,4-diphenyl-4,5-dihydro-1H-imidazol-1-yl]acetate Geometric parameters (Å, º)

**Table:**

C1—N1	1.476 (2)	C15—N1	1.274 (2)
C1—C8	1.531 (2)	C15—N2	1.406 (2)
C1—C14	1.539 (2)	C15—S1	1.7415 (18)
C1—C2	1.542 (2)	C16—S1	1.786 (2)
C2—C7	1.376 (3)	C16—H16A	0.9600
C2—C3	1.387 (3)	C16—H16B	0.9600
C3—C4	1.382 (3)	C16—H16C	0.9600
C3—H3	0.9300	C17—N2	1.445 (2)
C4—C5	1.373 (4)	C17—C18	1.501 (3)
C4—H4	0.9300	C17—H17A	0.9700
C5—C6	1.359 (4)	C17—H17B	0.9700
C5—H5	0.9300	C18—O2	1.189 (2)
C6—C7	1.394 (3)	C18—O3A	1.331 (2)
C6—H6	0.9300	C18—O3	1.331 (2)
C7—H7	0.9300	C19—O3	1.470 (3)
C8—C13	1.372 (3)	C19—C20	1.489 (5)
C8—C9	1.376 (3)	C19—H19A	0.9700
C9—C10	1.388 (3)	C19—H19B	0.9700
C9—H9	0.9300	C20—H20A	0.9600
C10—C11	1.347 (4)	C20—H20B	0.9600
C10—H10	0.9300	C20—H20C	0.9600
C11—C12	1.366 (4)	C19A—O3A	1.477 (5)
C11—H11	0.9300	C19A—C20A	1.489 (6)
C12—C13	1.390 (3)	C19A—H19C	0.9700
C12—H12	0.9300	C19A—H19D	0.9700
C13—H13	0.9300	C20A—H20D	0.9600
C14—O1	1.210 (2)	C20A—H20E	0.9600
C14—N2	1.371 (2)	C20A—H20F	0.9600
			
N1—C1—C8	109.77 (14)	S1—C16—H16A	109.5
N1—C1—C14	104.73 (13)	S1—C16—H16B	109.5
C8—C1—C14	113.33 (14)	H16A—C16—H16B	109.5
N1—C1—C2	110.96 (13)	S1—C16—H16C	109.5
C8—C1—C2	111.13 (14)	H16A—C16—H16C	109.5
C14—C1—C2	106.73 (14)	H16B—C16—H16C	109.5
C7—C2—C3	118.83 (18)	N2—C17—C18	113.00 (16)
C7—C2—C1	121.37 (17)	N2—C17—H17A	109.0
C3—C2—C1	119.76 (16)	C18—C17—H17A	109.0
C4—C3—C2	120.7 (2)	N2—C17—H17B	109.0
C4—C3—H3	119.6	C18—C17—H17B	109.0
C2—C3—H3	119.6	H17A—C17—H17B	107.8
C5—C4—C3	120.1 (2)	O2—C18—O3A	125.7 (2)
C5—C4—H4	119.9	O2—C18—O3	125.7 (2)
C3—C4—H4	119.9	O2—C18—C17	125.19 (19)
C6—C5—C4	119.5 (2)	O3A—C18—C17	109.14 (18)
C6—C5—H5	120.3	O3—C18—C17	109.14 (18)
C4—C5—H5	120.3	O3—C19—C20	105.4 (3)
C5—C6—C7	121.2 (2)	O3—C19—H19A	110.7
C5—C6—H6	119.4	C20—C19—H19A	110.7
C7—C6—H6	119.4	O3—C19—H19B	110.7
C2—C7—C6	119.7 (2)	C20—C19—H19B	110.7
C2—C7—H7	120.2	H19A—C19—H19B	108.8
C6—C7—H7	120.2	C19—C20—H20A	109.5
C13—C8—C9	118.12 (19)	C19—C20—H20B	109.5
C13—C8—C1	118.49 (17)	H20A—C20—H20B	109.5
C9—C8—C1	123.38 (17)	C19—C20—H20C	109.5
C8—C9—C10	120.8 (2)	H20A—C20—H20C	109.5
C8—C9—H9	119.6	H20B—C20—H20C	109.5
C10—C9—H9	119.6	C18—O3—C19	120.7 (3)
C11—C10—C9	120.6 (2)	O3A—C19A—C20A	104.0 (6)
C11—C10—H10	119.7	O3A—C19A—H19C	111.0
C9—C10—H10	119.7	C20A—C19A—H19C	111.0
C10—C11—C12	119.5 (2)	O3A—C19A—H19D	111.0
C10—C11—H11	120.2	C20A—C19A—H19D	111.0
C12—C11—H11	120.2	H19C—C19A—H19D	109.0
C11—C12—C13	120.4 (3)	C19A—C20A—H20D	109.5
C11—C12—H12	119.8	C19A—C20A—H20E	109.5
C13—C12—H12	119.8	H20D—C20A—H20E	109.5
C8—C13—C12	120.5 (2)	C19A—C20A—H20F	109.5
C8—C13—H13	119.7	H20D—C20A—H20F	109.5
C12—C13—H13	119.7	H20E—C20A—H20F	109.5
O1—C14—N2	125.35 (16)	C18—O3A—C19A	105.2 (5)
O1—C14—C1	129.75 (16)	C15—N1—C1	106.59 (14)
N2—C14—C1	104.84 (14)	C14—N2—C15	108.10 (14)
N1—C15—N2	115.62 (15)	C14—N2—C17	124.14 (15)
N1—C15—S1	127.39 (14)	C15—N2—C17	127.65 (15)
N2—C15—S1	116.97 (13)	C15—S1—C16	100.45 (10)
			
N1—C1—C2—C7	−3.0 (2)	C2—C1—C14—O1	62.8 (2)
C8—C1—C2—C7	−125.40 (19)	N1—C1—C14—N2	3.28 (17)
C14—C1—C2—C7	110.6 (2)	C8—C1—C14—N2	122.90 (15)
N1—C1—C2—C3	179.46 (17)	C2—C1—C14—N2	−114.45 (15)
C8—C1—C2—C3	57.0 (2)	N2—C17—C18—O2	−17.6 (3)
C14—C1—C2—C3	−67.0 (2)	N2—C17—C18—O3A	163.59 (16)
C7—C2—C3—C4	−0.5 (3)	N2—C17—C18—O3	163.59 (16)
C1—C2—C3—C4	177.1 (2)	O2—C18—O3—C19	−0.9 (4)
C2—C3—C4—C5	0.4 (4)	C17—C18—O3—C19	177.8 (2)
C3—C4—C5—C6	0.3 (4)	C20—C19—O3—C18	88.0 (5)
C4—C5—C6—C7	−0.9 (4)	O2—C18—O3A—C19A	9.4 (8)
C3—C2—C7—C6	−0.1 (3)	C17—C18—O3A—C19A	−171.8 (8)
C1—C2—C7—C6	−177.7 (2)	C20A—C19A—O3A—C18	162.3 (12)
C5—C6—C7—C2	0.8 (4)	N2—C15—N1—C1	0.2 (2)
N1—C1—C8—C13	−47.2 (2)	S1—C15—N1—C1	178.45 (13)
C14—C1—C8—C13	−163.92 (19)	C8—C1—N1—C15	−124.10 (15)
C2—C1—C8—C13	75.9 (2)	C14—C1—N1—C15	−2.12 (18)
N1—C1—C8—C9	133.98 (19)	C2—C1—N1—C15	112.68 (16)
C14—C1—C8—C9	17.3 (2)	O1—C14—N2—C15	179.40 (17)
C2—C1—C8—C9	−102.9 (2)	C1—C14—N2—C15	−3.19 (18)
C13—C8—C9—C10	−1.4 (3)	O1—C14—N2—C17	−4.2 (3)
C1—C8—C9—C10	177.42 (19)	C1—C14—N2—C17	173.26 (15)
C8—C9—C10—C11	−0.9 (4)	N1—C15—N2—C14	2.1 (2)
C9—C10—C11—C12	2.1 (4)	S1—C15—N2—C14	−176.36 (12)
C10—C11—C12—C13	−1.1 (5)	N1—C15—N2—C17	−174.20 (16)
C9—C8—C13—C12	2.3 (4)	S1—C15—N2—C17	7.4 (2)
C1—C8—C13—C12	−176.5 (2)	C18—C17—N2—C14	107.66 (19)
C11—C12—C13—C8	−1.1 (5)	C18—C17—N2—C15	−76.6 (2)
N1—C1—C14—O1	−179.47 (18)	N1—C15—S1—C16	4.8 (2)
C8—C1—C14—O1	−59.8 (2)	N2—C15—S1—C16	−176.94 (14)

### Ethyl 2-[2-(methylsulfanyl)-5-oxo-4,4-diphenyl-4,5-dihydro-1H-imidazol-1-yl]acetate Hydrogen-bond geometry (Å, º)

**Table:**

*D*—H···*A*	*D*—H	H···*A*	*D*···*A*	*D*—H···*A*
C5—H5···O2^i^	0.93	2.63	3.502 (3)	156
C9—H9···O1	0.93	2.40	3.063 (3)	128
C11—H11···O1^ii^	0.93	2.63	3.464 (3)	150
C17—H17*A*···O1^iii^	0.97	2.45	3.185 (2)	132

Symmetry codes: (i) *x*, *y*, *z*+1; (ii) *x*−1, *y*, *z*; (iii) −*x*+2, −*y*, −*z*+1.
